# PRmePRed: A protein arginine methylation prediction tool

**DOI:** 10.1371/journal.pone.0183318

**Published:** 2017-08-15

**Authors:** Pawan Kumar, Joseph Joy, Ashutosh Pandey, Dinesh Gupta

**Affiliations:** Translational Bioinformatics Group, ICGEB, New Delhi, India; Harbin Institute of Technology Shenzhen Graduate School, CHINA

## Abstract

Protein methylation is an important Post-Translational Modification (PTMs) of proteins. Arginine methylation carries out and regulates several important biological functions, including gene regulation and signal transduction. Experimental identification of arginine methylation site is a daunting task as it is costly as well as time and labour intensive. Hence reliable prediction tools play an important task in rapid screening and identification of possible methylation sites in proteomes. Our preliminary assessment using the available prediction methods on collected data yielded unimpressive results. This motivated us to perform a comprehensive data analysis and appraisal of features relevant in the context of biological significance, that led to the development of a prediction tool PRmePRed with better performance. The PRmePRed perform reasonably well with an accuracy of 84.10%, 82.38% sensitivity, 83.77% specificity, and Matthew’s correlation coefficient of 66.20% in 10-fold cross-validation. PRmePRed is freely available at http://bioinfo.icgeb.res.in/PRmePRed/

## Introduction

Protein arginine methylation (PRme) is an abundant post-translational modification (PTM) which affects several major cellular processes in eukaryotes. PRme has been implicated in several diseases and to such an extent that some eukaryotic viruses can take the liberty of host arginine methylation machinery for their own benefit. Any biological question which aims to investigate the role of PRme in a protein’s function, stability, localization and its interactions initiates with steps that lead to prior identification and validation of the methylation event. In this regard, large-scale proteomics, bolstered by recent advancements in PRme labeling, enrichment, and mass spectrometry (MS) techniques, have contributed significantly towards identification of experimentally verified repertoire of arginine methylated proteins. MS-based proteomics employing in vivo metabolic labeling of methyl group (Heavy- methyl SILAC) offers the best credible identification results as opposed to label-free approaches, which may be fraught poor reproducibility and discovery of artifact sites. However, apart from being expensive, metabolic labeling cannot be done for all biological samples such as intraerythrocytic *in vitro* cell culture of intracellular parasites like *P*. *falciparum*. Additionally, it is a tedious task to confirm each methylation site independently from the thousands of sites identified from a label-free MS experiment. Another option is to go for high throughput screening in vitro enzyme assays that use recombinant protein arginine methyltransferases (PRMTs; enzymes which catalyze arginine methylation) and protein/peptide substrates. However, any in vitro outcome cannot be considered as a natural event unless supported by in vivo evidence. Also, currently, one cannot perform such experiments for a very large number of arginine residues in any organism, for example nearly more than half a million arginine residues are present in human proteome (consisting of 20193 reviewed proteins from UniProt database [1] and excluding their isoforms) and that too with eleven different human PRMTs (again excluding their isoforms). Another limitation with any experiment involving a biological sample is that a cell, at any given time, usually never carries all the PTMs it can possibly acquire during its life cycle. Also, the specialized cell types in a multicellular organism produce their own distinct methylation profiles. Thus, due to several technical and analytical shortcomings, one cannot capture the entire spectrum of any particular PTM present in a cell/organism. Therefore, in such situations where it is difficult to perform reliable large-scale experimental studies for global PTM identification, one can use computational biology based approaches as an alternative strategy.

A computational tool called “FindMod” [2], which utilizes peptide mass fingerprinting data of individual proteins to identify methylated peptides, has been successfully applied in yeast proteome. However, this strategy has limited scope because it relies on peptide mass fingerprint (PMF) data of each protein which comes from single MS analysis and not tandem MS/MS. Therefore, reliability of assigned methylation sites is limited to only the peptides with no other PTM except methylation, and which only possess a single arginine and not any other amino acid capable of undergoing methylation (e.g. lysine) in their sequences. Another approach would be to find particular properties specific to methylation and use them to computationally identify potential PTM sites in whole proteomes. For example, one can employ a homology-based sequence search for evolutionarily conserved methylated sites present in evolutionarily conserved protein and domains. Histone proteins are highly conserved proteins in eukaryote kingdom; therefore, any characterized methylated arginine site in histone from one organism will most likely be methylated in other eukaryotes. Likewise, motif-based search can be employed if conserved motifs are known in the case of arginine methylation. In case of mammals particularly, it has been observed that several methylated sites lay in either glycine arginine-rich (GAR) or, arginine or proline-rich stretches. Unfortunately, there are no well-defined universal motifs in the case of arginine methylation. Hence, in such cases, machine learning based prediction models fits the choice of a universal method that can provide quick probing of large evolutionarily divergent proteomes to identify potential methylation sites. Consequently, fourteen machine-learning studies for prediction of arginine-methylated sites have been reported till date.

The first prediction tools developed by Daily et al. [3] and Shien et al. [4] introduced most of the key features that formed the backbone of the future methods. Subsequent tools focused more towards refinement of feature encoding, extraction and selection methods; resolving data imbalance and adoption of different classification approaches. The collection of arginine methylated sites employed by all the reported prediction based studies including the most recent ones, was restricted to few hundreds of methylation sites (about 200) which mostly were acquired from the UniProt database. A major surge in repertoire of identified arginine methylated sites came only post 2012 owing to several large-scale proteomic studies; however, these sites are yet to be incorporated into UniProt. Hence, we generated a database of the PRme data, which includes more than five thousand unique methylation sites. Of the 15 reported studies, only six provided access to user-friendly web server applications, whereas few others offer downloadable models, which unfortunately, we were unable to operate upon. Our preliminary assessment using each of the web server prediction application on our collected data yielded unimpressive results. This motivated us to perform our own comprehensive data analysis, appraisal of feature relevance in the context of biological significance (similar to Daily et al.) that led to the development of a prediction tool with better performance than the rest. We have also tried to offer an in-depth insight into the current problems faced in development PRme prediction methods, and possible areas of improvement.

Machine learning is a branch of artificial intelligence which has been successfully used for providing solutions to classification problems related to biological datasets. Amongst several machine learning algorithms, support vector machine (SVM), artificial neural networks (ANN), decision trees random forest (RF) and LibD3C are have been effectively used in bioinformatics [5–9]. Most of the available arginine methylation site prediction methods are based on SVMs, using different amino acid based features and feature selection methods. The training model developed in the study was trained on different machine learning algorithms for comparison and selection of the best training model for PRmePRed server.

## Methods

### Datasets for classifier generation

We collected experimentally verified *in vivo* methylated arginine sites from literature along with those reported in UniProt database (release 2015_06). Search terms like “arginine”, “methylation”, “methylation sites”, were used for database and literature searches. Peptides/proteins mentioned in the relevant publications (PubMed search performed in June-December 2015) were included in the study dataset only after close scrutiny. We did not consider any *in vitro* reported methylated sites with no credible evidence of *in vivo* existence. We removed sites/proteins with ambiguities such as those containing nonstandard amino acids, site mismatches, very small protein fragments (less than 30 aa) and obsolete protein entries. The extracted dataset contains 6754 methylation sites from 2077 protein sequences. We did not include any methylation sites from PhosphoSitePlus database [10], since it did not provide the exact experimental source and other supporting information for verifying PTM evidence. However, majority of our methylation data did match with the ones they reported to have extracted from the literature.

It is assumed that local environment around methylated arginine, dictated by adjacent flanking residues, plays a major role in substrate selectivity and catalysis by PRMTs. These assumptions arise from the observations in which PRMT active site and certain substrate features complement each other, though not always. For instance, in one substrate, positive flanking residues were shown to affect substrate binding and catalysis by PRMT active site [11]. This is supported by the fact that the surface surrounding active site in few PRMTs have grooves that are acidic in nature. Additionally, many of the known methylated arginine sites hail from either glycine-arginine rich (GAR) or arginine-rich and proline/serine-rich regions, which favor arginine methylation. In order to assess the role of flanking residues, we generated symmetric peptide datasets of varying window lengths (7, 11, 15, 19, 23, 27, 31 and 35) all of which were centered on methylated arginine. Since we adopted position specific feature encoding for model building, therefore it was necessary to fill the ends of peptides which lacked symmetry with arbitrary “X” residue that has been the generally accepted norm in some previous prediction classifiers as well [12].

We followed the conventional practice of generating a negative set from those sites which are not reported to be methylated in the methylated proteins. Briefly, we first created an unlabeled class of all the arginine sites, which are not methylated from the respective methylated proteins. We termed the set as unlabeled because they may contain potential sites, which could be methylated but has not been established yet. Using CD-HIT-2d [13] with 40% identity cut-off, we created a negative set from this unlabeled set by removing sequences which were similar to positive set.

There are chances that data will contain highly similar peptide sequences (since 2/3 of data belongs to human and mouse proteome, and also multiple adjacently placed arginine residues are methylated in sequences which are arginine rich such as those hailing from GAR peptides). Since most of our features are calculated position wise thus to reduce any biases especially during feature assessment with training set, we removed similar sequences from both positive and pseudo-negative sets using CD-HIT with 40% identity cut-off. We found that the pseudo-negative sets of window lengths 7, 11 and 15 were far lower than positive set and thus excluded from the model-building task. Dataset information (after CD-HIT) of different residues window length, chosen for model training are given in the supporting information S1 Table.

For each window length, positive dataset was split randomly into a training set and test set in the ratio of 4:1. We also split negative dataset into training and test set (size of the negative test set equal to positive test set). For window length 19 onward we had a larger proportion of negative training set with respect to a positive training set. Thus to overcome class imbalance issue we opted for under-sampling and created equal subsets of negative training set in 1:1 ratio with a positive training set by random sampling. For computational timesaving, we restricted the size of negative training subsets to 5 for each window length. During the course of our work, we accumulated more instances of arginine-methylated proteins from recent studies and separately prepared an independent dataset for final evaluation and comparison.

### Feature collection, encoding, and evaluation

An extensive literature survey implicated PRme with the amino acid composition; physico-chemical properties such as positive charge, hydrophilicity, isoelectric point; and structural properties including ASA and disorder. We finally collected the following features:

#### Atchley factors [14]

Since the distinct physico-chemical properties of amino acids reported in AAIndex [15] were too large to computationally handle in our analysis, therefore instead we relied on the reduced and transformed AAIndex feature subsets represented by the five Atchley factors (AF), namely, AF-I, AF-II, AF-III, AF-IV, and AF-V. Factor I represents residue polarity, hydrophobicity, and surface accessibility. Factor II captures secondary structure information whereas factor III relates to molecular size or volume. Factor IV reflects relative amino acid composition in various proteins and codon diversity. Factor V refers to electrostatic charge with high coefficients on isoelectric point and net charge. The PSE-in-One [16] features for protein are similar to AAIndex features, hence we did not consider them separately.

#### AA frequency

We generated amino acid composition features from position-wise amino acid frequency of each amino acid from the non-redundant positive peptide list. The values were normalized and a table of 21x n was created for each window, where n denotes window length.

#### ASA

ASA has been used as a feature by previous tools such as MASA [4] and PMeS [12], using RVP-NET for prediction of ASA values for amino acid residues, based on protein sequences. To evaluate the margin of error in these predictions, we compared the predicted values versus actual values calculated by NACCESS from PDB structures. For the sake of convenience, we considered only the methylated arginine sites from those protein sequences, which are represented by experimentally, solved PDB structures with greater than 80% sequence coverage and 100% identity.

#### Disorder [17]

Predicted protein intrinsic disorder was calculated for full length methylated protein sequences, using VSL2b standalone package. The output file for each protein sequence contained disorder scores for each residue. The predicted results of methylated proteins were compared with their respective experimental disorder information available in the DisProt database [18].

#### Hydrophobicity [19]

Hydrophobicity values for amino acids were obtained from Kyte and Doolittle hydrophobicity scales. The grand average of hydropathy (GRAVY) for a given peptide instance was calculated as sum average of hydrophobicity value of individual amino acids in the peptide.

#### Van der Waal’s volume

Van der Waal’s volume for each residue was calculated from scale reported by Darby and Creighton [20]. The average Van der Waals volume for each peptide was calculated as sum average of individual VDWV values.

#### Total charge and isoelectric point pI

Total charge and isoelectric point for each peptide were calculated using pyteomics, a python package [21].

For a given peptide instance, the following features Atchley factors, ASA, disorder, hydrophobicity, van der waal’s volume and AA frequency were encoded for individual residues in position wise manner whereas average VDWV, GRAVY, total charge, and pI were calculated for the entire peptide. Thus in total, we obtained feature sizes of 194, 234, 274, 314 and 354 for window lengths 19, 23, 27, 31 and 35 respectively.

Feature relevance assessment was performed by InfoGain (Information Gain) analysis on training sets in WEKA [22]. InfoGain selects the feature that has the best potential to separate the instances into individual classes. The value of InfoGain is lies between 0 and 1. A feature with a high information gain is said to be “relevant”. InfoGain is evaluated independently for each feature and the features with the top scores are selected as the relevant features.

The irrelevant features with a score of 0 were removed from total feature set and thus did not form part of feature selection. The removed features were indeed irrelevant as most of them belonged to a zeroth position which corresponded with central arginine thus corroborating that InfoGain analysis was correct. After removing irrelevant features (having value 0) from the total feature set, features set rearranged on the basis of relevance.

### Classifier

Support Vector Machines (SVMs), developed by Vladimir Vapnik and co-workers [23], is a useful technique for data classification. SVM is rigorously based on statistical learning theory. For linearly separable problems SVM employs a maximum margin hyper-plane for separating examples belonging to two different classes and for non-linearly separable problems, SVM first transforms the data into a higher dimensional feature space and subsequently employs a maximum margin linear hyper plane. There are four basic kernels that can be used in SVM.

$$\text{Linear}:\;\text{K}\left({\text{x}}_{\text{i}},\;{\text{x}}_{\text{j}}\right)\;=\;{\text{x}}^{\text{T}}{}_{\text{i}}{\text{x}}_{\text{j}}.$$

$$\text{Polynomial}:\;\text{K}\left({\text{x}}_{\text{i}},\;{\text{x}}_{\text{j}}\right)\;=\;{\left(\gamma {\text{x}}_{\text{i}}{}^{\text{T}}{\text{x}}_{\text{j}}+\;\text{r}\right)}^{\text{d}},\;\gamma \;>\;0.$$

$$\text{Radial}\;\text{basis}\;\text{function}\;\left(\text{RBF}\right):\;\text{K}\left({\text{x}}_{\text{i}},\;{\text{x}}_{\text{j}}\right)\;=\;\text{exp}\left(-\gamma ||{\text{x}}_{\text{i}}-{\text{x}}_{\text{j}}|{|}^{2}\right),\;\gamma \;>\;0.$$

$$\text{Sigmoid}:\;\text{K}\left({\text{x}}_{\text{i}},\;{\text{x}}_{\text{j}}\right)\;=\;\text{tanh}\left(\gamma {\text{x}}_{\text{i}}{}^{\text{T}}{\text{x}}_{\text{j}}+\;\text{r}\right).$$

Where, K(xi, xj) ≡ φ(x_i_)^T^ φ(x_j_) that is, the kernel function, represents a dot product of input data points mapped into the higher dimensional feature space by transformation.

Here, γ, r, and d are kernel parameters.

The RBF is by far the most popular choice of kernel types used in Support Vector Machines. This is mainly because RBF kernel non-linearly maps samples into a higher dimensional space so it, unlike the linear kernel, can handle the case when the relation between class labels and attributes is nonlinear.

LIBSVM (A Library for Support Vector Machines) [24] is currently one of the most widely used SVM software. A typical use of LIBSVM involves two steps: first, training a data set to obtain a model and second, using the model to predict information of a testing data set.

Here we used C-SVC from LIBSVM package with RBF kernel to build the classifier. C (cost) and g (gamma) optimized by grid search strategy using 10 fold cross validation with AUCROC as an evaluation function.

Major evaluation parameters: Accuracy (Acc), Sensitivity (Sn), Specificity (Sp) and Matthews Correlation Coefficient (MCC).

$$Sn=\frac{TP}{\text{TP}+\text{FN}}$$

$$Sp=\frac{TN}{\text{TN}+\text{FP}}$$

$$Acc=\frac{TP+TN}{\text{TP}+\text{FN}+\text{TN}+\text{FP}}$$

$$MCC=\frac{\left(\text{TP}\;\times \;\text{TN}\right)\;-\;\left(\text{FP}\;\times \;\text{FN}\right)}{\sqrt{\left(\text{TP}\;+\;\text{FP}\right)\;\left(\text{TP}\;+\;\text{FN}\right)\;\left(\text{TN}\;+\;\text{FP}\right)\;\left(\text{TN}\;+\;\text{FN}\right)}}$$

where, TP represents the number of correctly predicted methylated arginine sites by the SVM-predictor, TN represents the number of correctly predicted arginine non-methylated sites, FP represents the incorrectly predicted methylated arginine sites, and FN represents the incorrectly predicted arginine non-methylated sites. Further description of the terms is available elsewhere [25].

## Results and discussion

### Selection of feature subset and window size

Incremental feature selection was performed with various feature subsets in an incremental fashion for each window length. The evaluation parameters were compared with training data test and test set.

**Fig 1:**
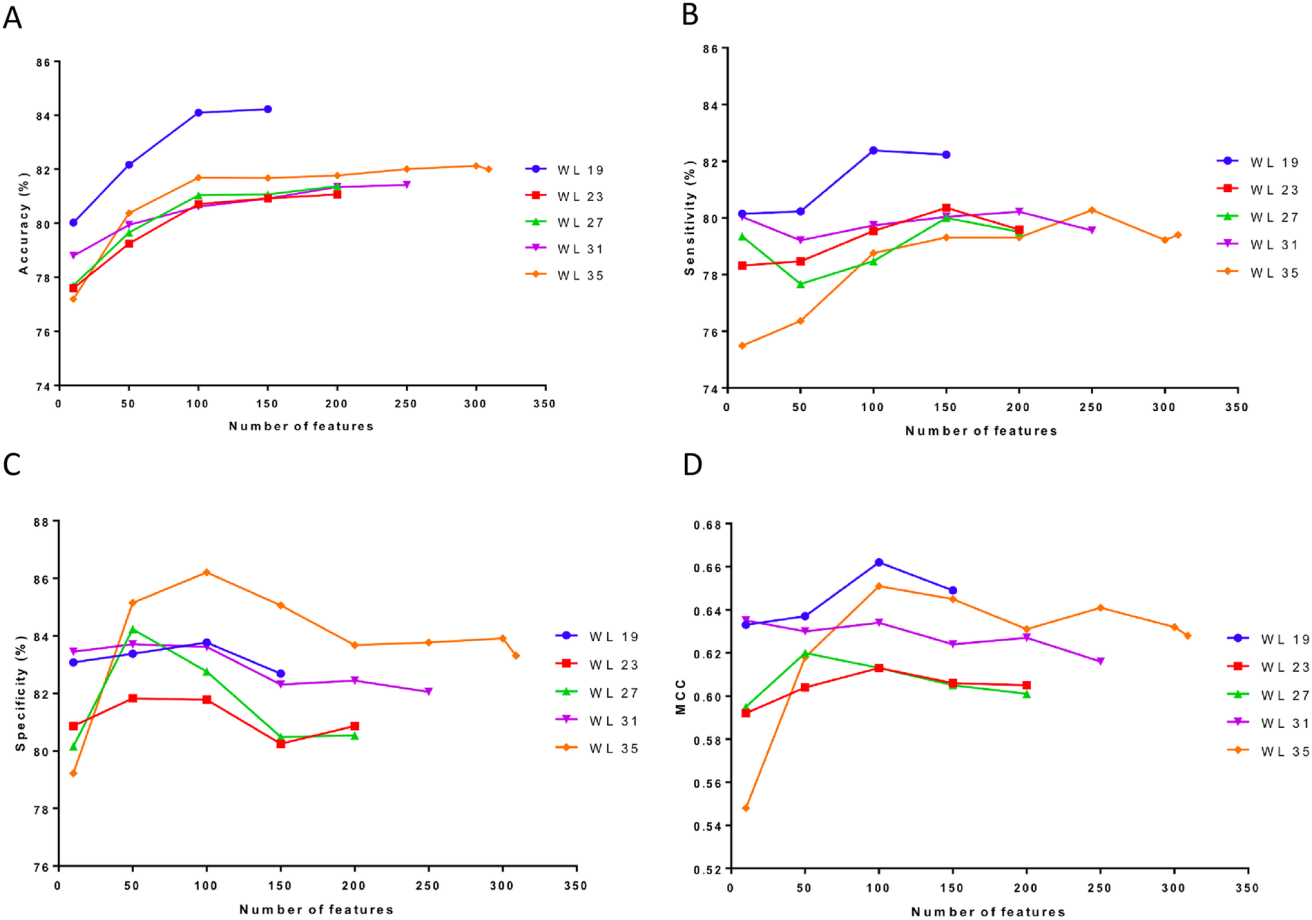
The relationship between different evaluation parameters and feature subsets. A) The relationship between the Accuracy and number of features. B) The relationship between the Sensitivity and number of features. C) The relationship between the Specificity and number of features. D) The relationship between the MCC and number of features.

For the arginine methylation prediction problem, best accuracy achieved by window length 19 with a subset of 150 features (Fig 1A), best sensitivity achieved by window length 19 with subset of 100 features (Fig 1B), best specificity achieved by window length 35 with subset of 100 features (Fig 1C) and the best MCC achieved by window length 19 with a subset of 100 features (Fig 1D). Considering all the evaluation parameters (Acc, Sn, Sp, and MCC), window length 19 with subsets of 100 features selected by information gain perform better (Table 1). The details of predictive performance of model trained with different features subset for window lengths 19, 23, 27, 31 and 35 may be obtained from supporting information S2–S6 Tables, respectively.

**Table 1:** Comparisons with best models of different window lengths.

Window Length (Features subset)	MCC	Accuracy	Sensitivity	Specificity
WL_19 (100)	0.662	84.10%	82.38%	83.77%
WL_23 (150)	0.606	80.93%	80.36%	80.25%
WL_27 (150)	0.605	81.07%	80.00%	80.49%
WL_31 (200)	0.629	81.35%	80.22%	82.45%
WL_35 (250)	0.641	82.01%	80.28%	83.77%

### Comparisons with existing methods

To further evaluate the prediction performance of the PRmePRed impartially, we made comparisons with other existing PRme prediction tools. Generally, to perform a comparison between distinct machine learning prediction methods, either cross-validation experiment or an independent dataset test is used. For cross-validation experiment, identical training dataset is required. As described in the Methods section PRmePRed training dataset is not similar to previous methods. Therefore, a comparison between distinct machine learning prediction methods through cross-validation performance is irrelevant. Here, we used independent dataset test to evaluate the performance of PRmePRed to compare it with other PRme prediction tools.

There are three major differences between our approach and previously reported methods. First, we used experimentally verified in vivo methylated arginine sites. Second to avoid any biases, we used CD-HIT (40%) on peptides rather than removing redundancy in protein sequences. Finally, rather than defining a broad range of parameters to describe the peptides, we used most relevant parameter for the methylation process. We used independent dataset to evaluate the performance of PRmePRed with comparison to other prediction tools (Table 2).

**Table 2:** Comparison of PRmePRed with other prediction methods.

Method (yr. developed)	Algorithm	MCC	Accuracy	Sensitivity	Specificity
MeMo (Chen et al. 2006)[26]	SVM	0.462	0.6839	0.3811	0.987
MASA (Shien et al. 2009)[4]	SVM	0.411	0.6503	0.3095	0.991
BPB-PPMS (Shao et al. 2009)[27]	SVM	0.253	0.5601	0.1202	1.000
PMeS (Shi et al. 2012)[12]	SVM	0.159	0.5756	0.4253	0.726
iMethyl-PseAAC (2014)[28]	SVM	0.302	0.5866	0.1768	0.997
PSSMe (Wen et al. 2016) [29]	SVM	0.444	0.7162	0.6003	0.832
MePred-RF (Wei et al. 2017) [30]	RF	0.462	0.6908	0.4095	0.972
PRmePRed (2017)	SVM	0.737	0.8683	0.8709	0.866

### ROC curve

**Fig 2:**
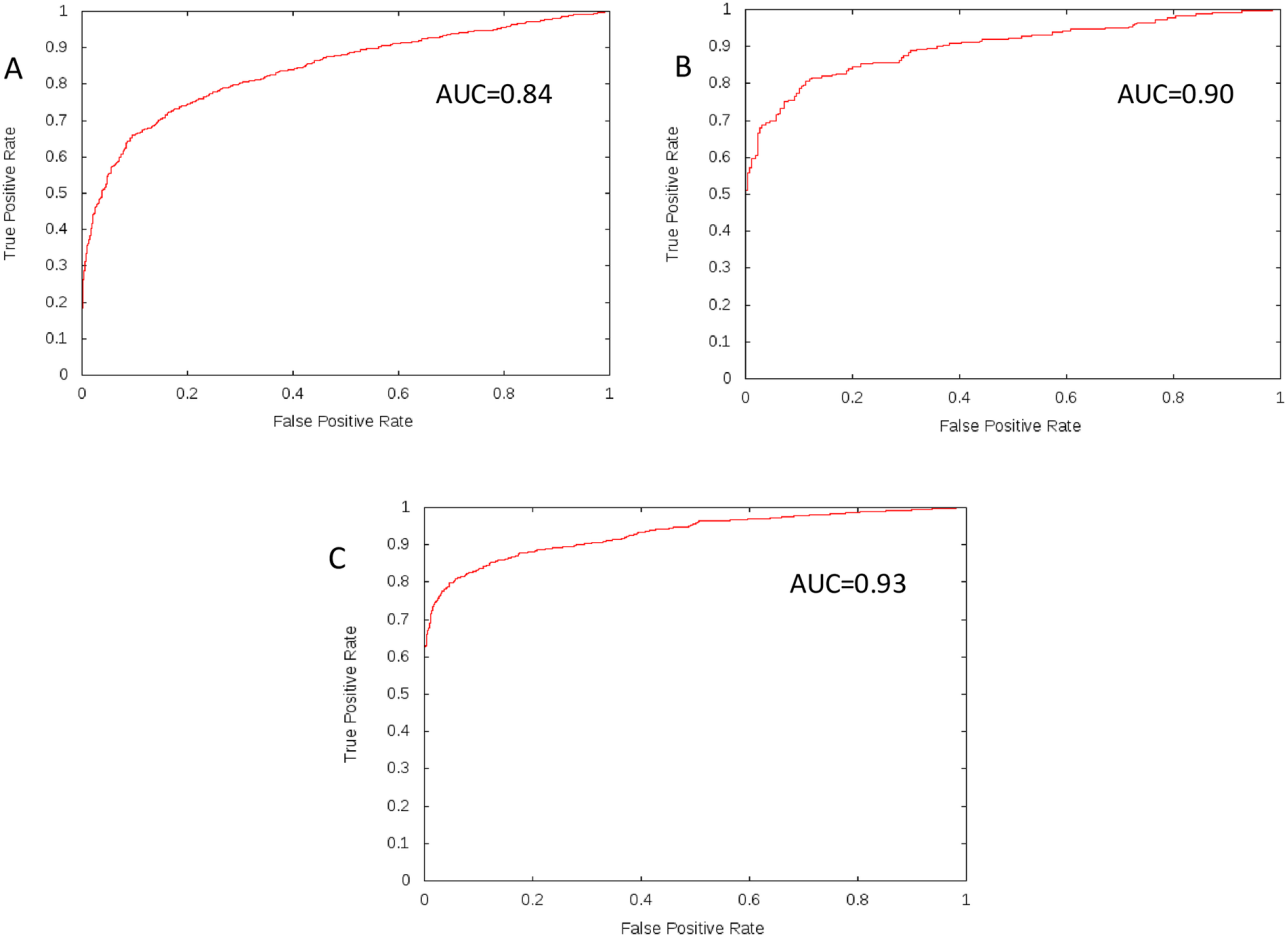
ROC curve for SVM classifier with different datasets. A) ROC curve for SVM classifier with training set. B) ROC curve for SVM classifier with test set. C) ROC curve for SVM classifier with independent set.

ROC curve is graphical display true positive rate (sensitivity) on y-axis and false positive rate (1 –specificity) on x-axis for varying cut-off points of test values. The area under the curve (AUC) is an effective and combined measure of sensitivity and specificity for assessing the inherent validity of a classification test. Maximum AUC = 1 and it means classification test is perfect in differentiating positive with negative class. This implies both sensitivity and specificity are one and both errors—false positive and false negative—are zero. This can happen when the distribution of methylated and non-methylated test values do not overlap. This is extremely unlikely to happen in practice. ROC curve of training model represent in Fig 2A (AUC = 0.8411), ROC curve of test data on training model represent in Fig 2B (AUC = 0.9000) and ROC curve of independent data on training model represent in Fig 2C (AUC = 0.9299). A result of 0.8 < = AUC < = 0.95 represent excellent ability to discriminate between of methylated and non-methylated arginine sites.

**Fig 3:**
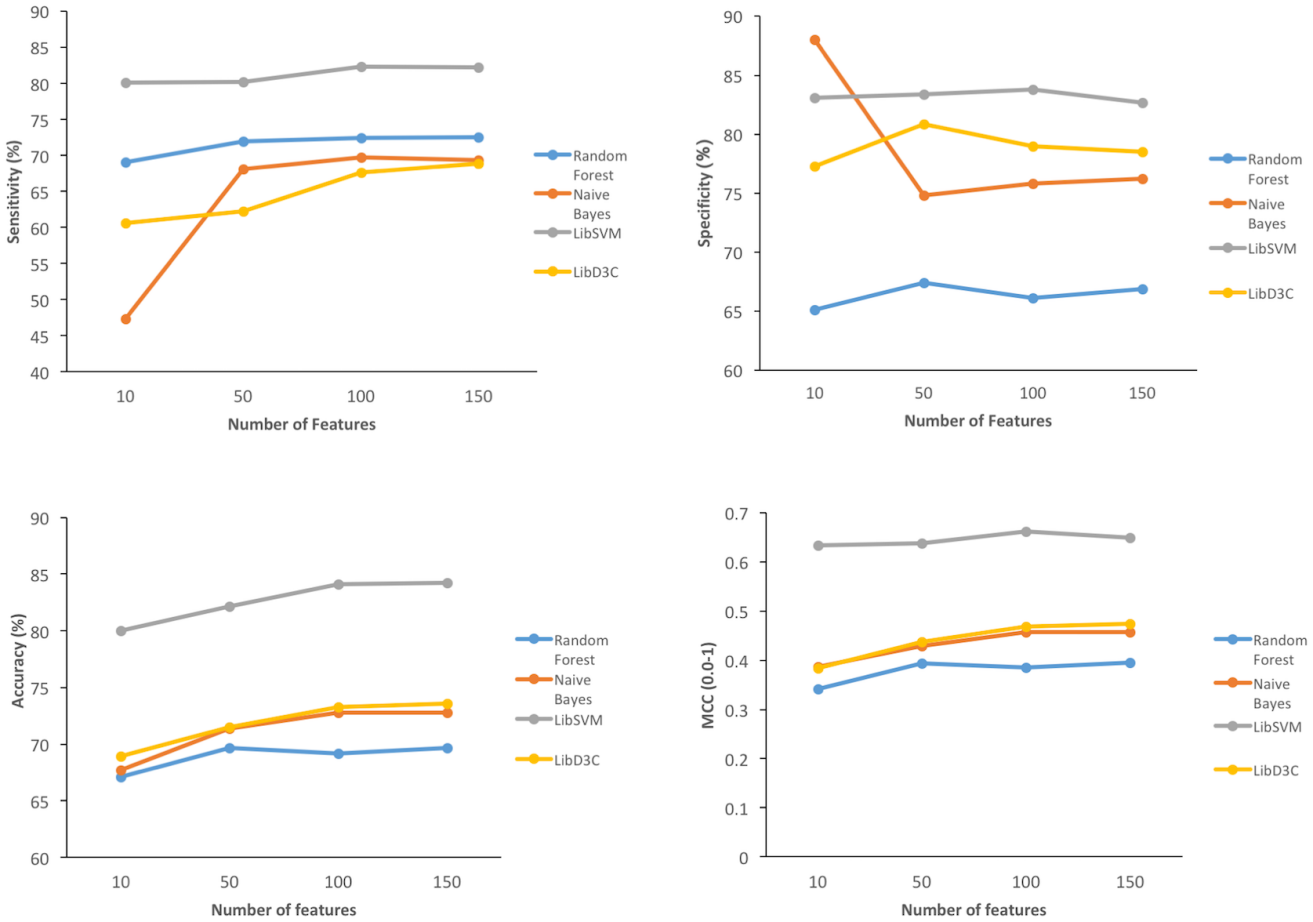
Comparisons with other classifiers based on evaluation parameters.

We evaluated SVM, RF, Naïve Bayes and LibD3C algorithms for PRmePRed development, and found that SVM performs comparatively better for the same set of features (See Fig 3).

## Conclusion

We have developed an arginine methylation predictor based on sequence and structure derived features, using SVMs. Dataset used to build the predictor is not biased and has experimentally verified entries only. Moreover, the PRmePRed shows better performance as compared with existing tools (Table 2). We believe that PRmePRed is a useful, reliable and rapid prediction tool for arginine methylation sites in proteins.

## Supporting information

S1 Table Dataset information of different residues window length. (DOC)

S2 Table The predictive performance of model trained with different features subset for window length 19. (DOC)

S3 Table The predictive performance of model trained with different features subset for window length 23. (DOC)

S4 Table The predictive performance of model trained with different features subset for window length 27. (DOC)

S5 Table The predictive performance of model trained with different features subset for window length 31. (DOC)

S6 Table The predictive performance of model trained with different features subset for window length 35. (DOC)
