# Supplementary material for: PRmePRed: A protein arginine methylation prediction tool
Source: PLoS One. 2017 Aug 15;12(8):e0183318. doi: 10.1371/journal.pone.0183318 (PMC5557562; doi:10.1371/journal.pone.0183318)
Supplement: S1 Table — (DOC) [file pone.0183318.s001.doc]

**Table S1.** Dataset information (after CD-HIT) of different residues window length

|  |  | Positive Dataset |  |  | Negative Dataset |  |
| --- | --- | --- | --- | --- | --- | --- |
| Window Length | Complete  Set | Training Set (80%) | Test Set (20%) | Complete  Set | Training Set | Test Set |
| 19 | 1298 | 1038 | 260 | 5539 | 5279 | 260 |
| 23 | 1964 | 1571 | 393 | 20004 | 19611 | 393 |
| 27 | 1845 | 1476 | 369 | 17729 | 17360 | 369 |
| 31 | 2288 | 1830 | 458 | 31603 | 31145 | 458 |
| 35 | 2175 | 1740 | 435 | 28250 | 27815 | 435 |
