# Supplementary material for: PRmePRed: A protein arginine methylation prediction tool
Source: PLoS One. 2017 Aug 15;12(8):e0183318. doi: 10.1371/journal.pone.0183318 (PMC5557562; doi:10.1371/journal.pone.0183318)
Supplement: S2 Table — (DOC) [file pone.0183318.s002.doc]

**Table S2. The predictive performance of model trained with different features subset for window length 19**

| Features number | Accuracy | Sensitivity | Specificity | MCC |
| --- | --- | --- | --- | --- |
| 10 | 80.03% | 80.15% | 83.08% | 0.633 |
| 50 | 82.17% | 80.23% | 83.38% | 0.637 |
| 100 | 84.10% | 82.38% | 83.77% | 0.662 |
| 150 | 84.23% | 82.23% | 82.69% | 0.649 |
