# Supplementary material for: PRmePRed: A protein arginine methylation prediction tool
Source: PLoS One. 2017 Aug 15;12(8):e0183318. doi: 10.1371/journal.pone.0183318 (PMC5557562; doi:10.1371/journal.pone.0183318)
Supplement: S3 Table — (DOC) [file pone.0183318.s003.doc]

**Table S3. The predictive performance of model trained with different features subset for window length 23.**

| Features number | Accuracy | Sensitivity | Specificity | MCC |
| --- | --- | --- | --- | --- |
| 10 | 77.60% | 78.32% | 80.87% | 0.592 |
| 50 | 79.25% | 78.47% | 81.83% | 0.604 |
| 100 | 80.72% | 79.54% | 81.78% | 0.613 |
| 150 | 80.93% | 80.36% | 80.25% | 0.606 |
| 200 | 81.07% | 79.59% | 80.87% | 0.605 |
