# Supplementary material for: PRmePRed: A protein arginine methylation prediction tool
Source: PLoS One. 2017 Aug 15;12(8):e0183318. doi: 10.1371/journal.pone.0183318 (PMC5557562; doi:10.1371/journal.pone.0183318)
Supplement: S4 Table — (DOC) [file pone.0183318.s004.doc]

**Table S4. The predictive performance of model trained with different features subset for window length 27.**

| Features number | Accuracy | Sensitivity | Specificity | MCC |
| --- | --- | --- | --- | --- |
| 10 | 77.70% | 79.35% | 80.16% | 0.595 |
| 50 | 79.66% | 77.67% | 84.23% | 0.620 |
| 100 | 81.04% | 78.48% | 82.76% | 0.613 |
| 150 | 81.07% | 80.00% | 80.49% | 0.605 |
| 200 | 81.38% | 79.51% | 80.54% | 0.601 |
