# Supplementary material for: PRmePRed: A protein arginine methylation prediction tool
Source: PLoS One. 2017 Aug 15;12(8):e0183318. doi: 10.1371/journal.pone.0183318 (PMC5557562; doi:10.1371/journal.pone.0183318)
Supplement: S5 Table — (DOC) [file pone.0183318.s005.doc]

**Table S5. The predictive performance of model trained with different features subset for window length 31.**

| Features number | Accuracy | Sensitivity | Specificity | MCC |
| --- | --- | --- | --- | --- |
| 10 | 78.80% | 80.04% | 83.45% | 0.635366741 |
| 50 | 79.94% | 79.21% | 83.71% | 0.6299689 |
| 100 | 80.62% | 79.74% | 83.62% | 0.634183603 |
| 150 | 80.92% | 80.04% | 82.31% | 0.623858896 |
| 200 | 81.35% | 80.22% | 82.45% | 0.626799275 |
| 250 | 81.42% | 79.56% | 82.05% | 0.616379514 |
