# Supplementary material for: PRmePRed: A protein arginine methylation prediction tool
Source: PLoS One. 2017 Aug 15;12(8):e0183318. doi: 10.1371/journal.pone.0183318 (PMC5557562; doi:10.1371/journal.pone.0183318)
Supplement: S6 Table — (DOC) [file pone.0183318.s006.doc]

**Table S6. The predictive performance of model trained with different features subset for window length 35.**

| Features number | Accuracy | Sensitivity | Specificity | MCC |
| --- | --- | --- | --- | --- |
| 10 | 77.20% | 75.49% | 79.22% | 0.548 |
| 50 | 80.38% | 76.37% | 85.15% | 0.618 |
| 100 | 81.69% | 78.76% | 86.21% | 0.651 |
| 150 | 81.68% | 79.31% | 85.06% | 0.645 |
| 200 | 81.77% | 79.31% | 83.68% | 0.631 |
| 250 | 82.01% | 80.28% | 83.77% | 0.641 |
| 300 | 82.13% | 79.22% | 83.91% | 0.632 |
| 309 | 82.00% | 79.40% | 83.31% | 0.628 |
